# Coordinated mitochondrial quality control underlies oxidative stress resilience in MUSE stem cells

**DOI:** 10.1080/13510002.2026.2698189

**Published:** 2026-10-09

**Authors:** Domenico Aprile, Sura Hilal Ahmed Al-Sammarraie, Alessia Ambrosino, Mohd Shahzaib, Yesuf Siraj, Afshin Samiminemati, Nicola Alessio, Gianfranco Peluso, Giovanni Di Bernardo, Umberto Galderisi

**Affiliations:** a Department of Life Sciences, Health and Health Professions, Link Campus University, Rome, Italy; b Department of Experimental Medicine, Luigi Vanvitelli Campania University, Naples, Italy; c Department of Biotechnology, University of Samarra, Samarra, Iraq; d Department of Medical Laboratory Sciences, Bahir Dar University, Bahir Dar, Ethiopia; e Saint Camillus International University of Health and Medical Sciences, UNICAMILLUS, Rome, Italy; f Genome and Stem Cell Center (GENKÖK), Erciyes University, Kayseri, Turkey

**Keywords:** MUSE stem cells, reactive oxygen species, oxidative stress, mitochondrial quality control, mitochondrial DNA damage, mitophagy, mitochondrial biogenesis, PGC-1α, NRF2

## Abstract

**Background:**

Reactive oxygen species (ROS) regulate stem-cell function, but sustained exposure causes oxidative damage, mitochondrial dysfunction and exhaustion. Multilineage-differentiating Stress-Enduring (MUSE) cells, a rare SSEA-3⁺ MSC subset, tolerate ROS-rich environments, although the mechanisms remain unclear.

**Methods:**

We compared MUSE, Non-MUSE (SSEA-3⁻) and MSCs after H₂O₂ exposure, assessing ROS clearance, mtDNA integrity, 8-oxo-dG, antioxidant and BER responses, mitochondrial morphology/function, mitophagy and biogenesis, with focus on the PGC-1α/NRF2 axis. Three independent biological replicates were analyzed by imaging, qPCR, flow cytometry and biochemical assays.

**Results:**

MUSE cells showed greater resistance to oxidative stress than MSCs and Non-MUSE cells. After H₂O₂, they displayed lower ROS accumulation (~15–25%), preserved mtDNA integrity, reduced 8-oxo-dG, rapid induction of antioxidant and BER-related genes, and maintenance of mitochondrial morphology, membrane potential and respiratory activity. MUSE cells also showed enhanced mitophagy, with >2-fold increased LAMP1–MitoTracker colocalization and induction of PINK1/phospho-Ub-S65, together with increased biogenesis, including ~45% higher COX-I/SDHA ratio, TFAM induction and PGC-1α activation at 48 h.

**Conclusion:**

MUSE cells coordinate antioxidant defense, DNA repair, mitophagy and biogenesis through a remove-and-replace program that preserves mitochondrial function during oxidative stress.

## Graphical abstract

**Figure:**
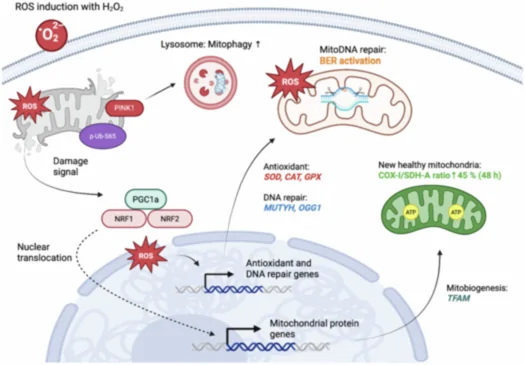

Proposed model of mitochondrial quality control in MUSE stem cells for stress endurance.

Mitochondrial ROS accumulation activates the PINK1/p-Ub-S65 pathway, initiating mitophagy to remove damaged organelles. Concurrently, oxidative stress promotes nuclear translocation of PGC1α and activation of the NRF1/NRF2 transcriptional axis, leading to upregulation of: (1) antioxidant genes (*SOD1, CAT, GPX1*); (2) DNA repair genes (MUTYH, OGG1); and (3) mitochondrial proteins. The base excision repair (BER) pathway is activated to repair mtDNA damage, while TFAM mediates mitochondrial biogenesis, ultimately generating new healthy mitochondria as evidenced by the increased COX-I/SDH-A ratio at 48 hours post-stress (generated with BioRender).

## Introduction

Tissue regeneration is initiated by a cascade of signals released by stem cells within the niche immediately after injury. Damaged parenchymal and immune cells secrete pro-inflammatory cytokines (e.g. TNF-α, IL-1β, IL-6) and generate bursts of reactive oxygen species (ROS) that serve as early ‘danger’ messengers to activate resident stem and progenitor cells [1,2]. ROS act according to a biphasic, concentration-dependent paradigm: moderate levels operate as second messengers that enhance proliferation, migration and lineage specification, whereas sustained concentrations oxidise macromolecules, collapse mitochondrial bioenergetics and ultimately exhaust the stem-cell pool [3,4]. Hence, regenerative success depends on each stem-cell type's ability to sense, buffer and adapt to fluctuating oxidative loads.

Mitochondria lie at the centre of this adaptive response. In quiescent stem cells, mitochondrial content is minimal, oxidative phosphorylation (OXPHOS) is repressed, and antioxidants maintain a highly reduced intracellular milieu. Upon activation, cells must reprogramme their mitochondrial network, increasing biogenesis, modulating fusion–fission cycles, and engaging selective mitophagy, to meet new energetic requirements while preventing excessive ROS build-up [5–7]. Evidence from diverse systems underscores the importance of this plasticity: muscle satellite cells tolerate transient ROS spikes by synchronously up-regulating PGC-1α-driven biogenesis and Parkin-dependent mitophagy [8]; neural stem cells shift from glycolysis to OXPHOS while activating NRF2 in response to hypoxic stress [9]; conversely, haematopoietic stem cells with weaker antioxidant capacity and slower mitochondrial turnover accumulate oxidative DNA lesions and undergo functional attrition [3].

These observations highlight a broader principle: differential ROS tolerance is an evolutionary trade-off between repair speed, genome protection and cancer risk. Tissues frequently exposed to mechanical or metabolic stress (e.g. skeletal muscle, skin) select for stem-cell pools equipped with more flexible mitochondrial quality-control (MQC) programmes; long-lived, slowly cycling compartments (e.g. bone marrow) favour strict redox restraint to minimise mutagenesis [10].

From a translational standpoint, understanding how highly stress-resilient stem cell subsets regulate mitochondrial quality control may provide useful biological insight into the determinants of stem cell survival under oxidative challenge. Rather than addressing therapeutic application directly, dissecting the MQC logic of these cells helps clarify the cellular strategies that enable rapid adaptation to fluctuating redox conditions. Such mechanistic knowledge may, in the longer term, inform the rational development of experimental approaches aimed at enhancing stress tolerance in less resilient cell populations.

Mesenchymal stromal cells (MSCs), which comprise a subset of stem cells, exemplify a population capable of orchestrating tissue repair at a distance. Following systemic or local administration, they release a complex secretome enriched with cytokines, growth factors, lipid mediators, and extracellular vesicles carrying various macromolecules such as miRNAs. These factors modulate immune responses, angiogenesis, and fibrosis well beyond the site of implantation [11,12]. This paracrine pharmacology has underpinned >1000 clinical trials in cardiology, orthopaedics and immune-mediated disorders. Yet bulk MSC preparations are heterogeneous and only moderately stress-resistant. Within them, ~1%–3% of adherent cells express the stage-specific embryonic antigen-3 (SSEA-3). These Multilineage-differentiating Stress-Enduring (MUSE) stem cells are non-tumorigenic, self-renew spontaneously and differentiate into derivatives of all three germ layers without forming teratomas [13–15]. Crucially, MUSE cells home selectively to damaged sites: mobilised from reservoirs such as bone marrow and dermis, they follow sphingosine-1-phosphate and other injury cues, engraft, survive the hostile micro-environment better than bulk MSCs, and can replace lost cells with mature, tissue-specific cells [16–18].

Notably, we have previously demonstrated that this superior stress endurance extends to apoptotic resistance: MUSE cells exhibit markedly reduced activation of executioner caspases and sustain lower levels of DNA fragmentation compared to parental MSCs when exposed to serum deprivation or hypoxic injury [19,20].

Although their migratory and paracrine abilities are well documented, the mitochondrial mechanisms that permit MUSE cells to thrive in ROS-rich lesions remain unexplored.

In this study, we ask whether MUSE cells achieve their extraordinary stress resilience by synchronising all four legs of mitochondrial quality control: DNA preservation, balanced fusion–fission, PINK1/Parkin-mediated mitophagy and PGC-1α/NRF2-driven biogenesis, into a single, rapid-response circuit [5,21,22]. By comparing MUSE cells with their SSEA-3⁻ siblings and parental MSCs under an acute oxidative challenge, we test the overarching hypothesis that only the SSEA-3⁺ fraction can couple swift removal of damaged organelles to equally prompt replacement, thereby safeguarding bioenergetics and genomic integrity. Clarifying this coordination will fill a critical gap in our understanding of stem-cell heterogeneity in ROS handling and may help clarify how distinct stem cell subsets adapt to oxidatively hostile microenvironments at the cellular level.

## Materials and methods

### Cell culture

Normal human bone marrow–derived mesenchymal stromal cells (hMSCs; ATCC, Cat. PCS-500-012™, RRID:CVCL_1D56) were obtained in [March 2025; Lot 80811242] and expanded according to the manufacturer's instructions. Cultures were routinely tested and confirmed negative for mycoplasma contamination (MycoAlert™, Lonza, LT07-318). In detail, the cells after thawing were cultivated in proliferating medium: DMEM low glucose medium (Gibco, 11885084, Thermo Fisher Scientific) containing 10% ES-FBS (Euroclone Spa, ECS0180L), 4 mM L-glutamine (Gibco, 25030081, Thermo Fisher Scientific), 100 U/ml penicillin–streptomycin (Gibco, 15140122, Thermo Fisher Scientific) and 3 ng/ml bFGF2 (PeproTech, Cat. No. # 100-18B-50UG). The cells were incubated at 37 °C in a humidified, 5% CO_2_ atmosphere until the fourth passage (P4).

#### MUSE cells isolation from MSCs

Confluent MSCs at P4 were collected by 0.25% trypsin–EDTA (Sigma, T4049, Thermo Fisher Scientific) and were subjected to cell sorting to isolate MUSE cells. MSCs were suspended in MACS buffer, which contained 0.5% bovine serum albumin (BSA) (Sigma, Cat. No. A7906) and 2 mM EDTA (Sigma, Cat. No. E9884) in FluoroBrite DMEM (Gibco, A1896701), and were incubated with the anti-human SSEA-3 antibody (Biolegend, 330302) for 1 h on ice. Cells were then washed three times with MACS buffer and centrifuged at 400g for 5 min. Afterwards, cells were incubated with a secondary antibody, anti-rabbit IgM-FITC (ImmunoReagents, RbxHu-006-DFITC) for 1 h on ice and subsequently washed three times again. Cells were then incubated with anti-FITC microbeads (Miltenyi, 130-097-050) for 15 min on ice and later washed with MACS buffer. Magnetic-activated cell sorting (MACS) was used to collect SSEA-3(+) cells and SSEA-3(−) non-MUSE cells according to the manufacturer's instructions (Miltenyi). The collected SSEA-3 positive cells represented MUSE cells and were cultivated in suspension on poly-2-hydroxyethyl methacrylate (pHEMA) (Sigma, 192066) coated petri dishes in a complete medium composed of DMEM low glucose medium (Gibco, 11885084) containing 10% ES-FBS (Euroclone Spa), 4 mM L-glutamine (Gibco), 100 U/ml penicillin–streptomycin (Gibco), 3 ng/ml bFGF2 (PeproTech) and 2.6% MethoCult (STEMCELL Technologies, Catalogue # 04100) for 7 days. At the end, the cells were plated for successive experiments or subjected to analysis [23,24]. Sorted populations were analysed by flow cytometry (BD Accuri C6 Plus) using anti-SSEA-3-PE (BioLegend, 330103, 1:50) to confirm the correct isolation and enrichment of the respective cell populations. All experiments were performed after prospective isolation of SSEA3⁺ MUSE cells and SSEA3^−^ Non-MUSE cells.

#### Oxidative-stress induction

Cells were seeded at 5 × 10^4^ cells cm^2^ and, after overnight attachment, exposed to 300  µM H₂O₂ (Sigma, H1009) for 30 min at 37 °C. Cultures were then washed twice with PBS and incubated in fresh medium for 1 h, 24 h, or 48 h prior to downstream assays. The 300 μM H₂O₂ pulse for 30 min was selected based on preliminary titration experiments and previous literature showing that this concentration induces transient oxidative stress without causing massive acute cell death in MSC-like populations [25,26].

#### Pharmacological inhibition of PGC1α and NRF2

For pathway studies, cells were pre-treated for 16 h with either 10 µM SR-18292 (PGC1α inhibitor, SelleckChem, S8696) or 5 µM ML385 (NRF2 inhibitor, Sigma, SML1833). Treatments were maintained during the subsequent H₂O₂ challenge. Vehicle controls received 0.1% DMSO.

#### Subcellular fractionation

Mitochondrial and nuclear enrichment was performed according to Liao's protocol [27]. The cell pellet obtained from cultured cells treated as previously described (approximately 2 × 10⁶ cells) was resuspended in five volumes of extraction buffer (0.25 M sucrose, 20 mM HEPES–KOH, pH 7.5, 10 mM KCl, 1.5 mM MgCl₂, 1 mM EDTA, 1 mM EGTA, 1 mM dithiothreitol, and 0.1 mM PMSF, all reagents were from Sigma). The resulting cell suspension was homogenized using a Teflon-glass homogenizer by performing 20 up-and-down strokes with the pestle. Nuclei and mitochondria were then separated through differential centrifugation following the established protocol.

Cytoplasmic and nuclear protein fractions were subsequently prepared with the classic ‘mini-extract’ method of Schreiber and collaborators [28]. Briefly, cell pellets were gently swollen for 10 min in ice-cold hypotonic buffer (10 mM HEPES-KOH pH 7.9, 10 mM KCl, 1.5 mM MgCl₂, 0.5 mM DTT, protease/phosphatase inhibitors) and disrupted by the addition of 0.1% NP-40 followed by 10 strokes in a tight Dounce homogeniser. Nuclei were pelleted (800 g, 5 min, 4 °C); the supernatant constituted the cytosolic extract. Nuclear pellets were washed once in hypotonic buffer and extracted for 20 min on ice in high-salt buffer (20 mM HEPES-KOH, pH 7.9, 420 mM NaCl, 25% glycerol, 1.5 mM MgCl₂, 0.5 mM DTT, inhibitors). Insoluble debris was removed (15,000 g, 10 min, 4 °C) and the clarified supernatant collected as the nuclear extract.

Fraction purity was verified by western blot for MT-ND4 (mitochondria), H4 (nuclei) and GAPDH (cytosol) (Sup. Figure S2). All information regarding antibodies and dilutions is reported in Supplementary Table 1.

### Measurement of intracellular ROS

The generation of ROS was monitored by the conversion of fluorogenic 2,7-dichlorodihydrofluorescein diacetate (DCFH-DA) to highly fluorescent dichlorofluorescein diacetate within cells by ROS. The cells were incubated with 2 μM DCFH-DA (Sigma, 35845) in CO_2_ Independent Medium (Gibco) and acquired every hour for 48 h on a microplate reader (GloMax® Discover, Promega) with ex-em 495–525 nm. DCFH-DA fluorescence was normalized to baseline fluorescence.

### Measurement of mitochondrial ROS accumulation

Mitochondrial superoxide accumulation was evaluated using the Mitochondrial Superoxide Assay Kit (Fluorometric) (Abcam, ab219943), according to the manufacturer's instructions. Briefly, MSCs, MUSE, and Non-MUSE cells were seeded in black-wall 96-well plates and exposed to 300 μM H₂O₂. One hour after oxidative stress induction, cells were incubated with the mitochondrial superoxide-sensitive fluorescent probe provided in the kit for 30 min. Fluorescence was measured using a microplate reader (GloMax® Discover, Promega) at ex/em = 540/590 nm.

### Mitochondrial DNA integrity

Mitochondrial DNA (mtDNA) damage was evaluated by long-amplicon quantitative PCR adapted from the gene-specific QPCR method of Kovalenko & Santos [29–31]. Total cellular DNA containing mitochondrial DNA (20 ng) was amplified with DNA-polymerase (Promega). A 8.9 kb fragment spanning positions 5999–14,841 of the human mtDNA genome was amplified for 30 cycles (95 °C 45 s, 65 °C 45 s, 72 °C 12 min) using a hot-start step at 95 °C for 5 min. A 221 bp mtDNA locus served as an amplification control to normalise for copy number. Amplicons were resolved on 0.7% agarose gels, stained with GelStar and imaged under non-saturating conditions; band densities were quantified with ChemiDoc (Biorad). Quantification was performed by the ratio of big/small mito fragments.

### Base-excision-repair (BER) activity

Uracil-DNA glycosylase (UDG)-dependent BER activity was quantified according to Squillaro et al., with minor adaptations [32]. Briefly, a 327-bp fragment of the human GAPDH gene was amplified by PCR using a nucleotide mixture in which dUTP was incorporated into the PCR product. The primers used to generate the substrate were: forward 5′-GCATCCTGCACCACCAACTG-3′ and reverse 5′-GCCTGCTTCACCACCTTCTT-3′. The resulting dUTP-containing GAPDH amplicon was used as a DNA substrate for the in vitro repair/incision reaction. Mitochondrial or nuclear protein extracts (20 µg) were incubated for 30 min at 37 °C with the dUTP-containing substrate (150 ng) in 20 µL of reaction buffer (70 mM HEPES-KOH pH 7.5, 1 mM EDTA, 1 mM DTT, 75 mM NaCl, 0.5% BSA). The reaction was terminated with 0.2% SDS and 2 µg Proteinase K (55 °C, 15 min)(sigma, P2308-5MG), followed by phenol–chloroform extraction and ethanol precipitation. Purified DNA were amplified with BrightGreen 2× qPCR MasterMix (ABM, G891) on a LineGene 9600 machine (Bioer Technology) using primers flanking the lesion site (forward 5′-GCATCCTGCACCACCAACTG-3′ and reverse 5′-GCCTGCTTCACCACCTTCTT-3′). Cycling: 95 °C 30 s, then 40 cycles of 95 °C 5 s, 58 °C 30 s. BER activity was quantified by calculating the number of successfully repaired DNA molecules based on a standard curve generated from known concentrations of template DNA. A reaction containing 0.1 U AmpErase UDG (Thermo Fisher, Cat. No. N8080096) served as a positive control; reactions without uracil residues served as negative controls. In this assay, UDG activity present in the extracts recognizes and removes uracil residues from the DNA substrate, thereby reducing the amount of intact template available for subsequent qPCR amplification.

### Quantitative RT-qPCR

Total RNA was extracted from cell cultures using a RNeasy Mini Kit (Qiagen, 74104) following the manufacturer's instructions. The quality and quantity of isolated RNA were analysed spectrophotometrically using a Nanodrop (Thermo Fisher). We used sequences of mRNAs from the Nucleotide Data Bank (National Center for Biotechnology Information) to design primer pairs for real-time RT-qPCR reactions (Primer Express v. 3.0, Applied Biosystems). The primer sequences are reported in Supplementary Table 2. cDNA synthesis was performed by 5× ALL-INONE RT MASTERMIX (ABM, G592). We used appropriate regions of GAPDH cDNA as controls and ran real-time PCR assays on a Line- Gene 9600 machine (Bioer Technology). We carried out reactions according to the manufacturer's instructions using a BrightGreen 2× qPCR MasterMix (ABM, G891). We used the 2^−ΔΔCT^ method as a relative quantification strategy for quantitative real-time PCR data analysis.

### Mitochondrial quantification and morphology analysis

Live cells were stained with 200  nM MitoTracker™ Red CMXRos (Invitrogen, M7512) for 30 min, fixed with 2% formaldehyde, and imaged on a Zeiss LSM-880. Mean fluorescence intensity (MFI) of MitoTracker signal was quantified using ImageJ/Fiji. Briefly, raw images were converted to 8-bit and a uniform threshold was applied to improve mitochondrial structure definition and reduce background fluorescence. Regions of interest (ROIs) corresponding to whole cells were manually outlined, and the integrated density (sum of pixel values within the ROI) was measured. Mean fluorescence intensity was then calculated as integrated density divided by the ROI area. At least 300 cells per condition were analyzed from 3 independent experiments. Mitochondrial length and form-factor were quantified in ≥300 cells per condition using the ‘Mitochondria Analyzer’ ImageJ plug-in [33,34].

### Measurement of mitochondrial respiratory activity (OCR)

Mitochondrial respiratory activity was evaluated using an Enhanced Oxygen Consumption Rate (OCR) Fluorometric Assay Kit (Elabscience E-BC-F070) performed in 96-well plates according to the manufacturer's instructions. Briefly, MSCs, MUSE, and Non-MUSE cells were seeded in black-wall 96-well plates and exposed to 300 μM H₂O₂. Twenty-four hours after oxidative stress induction, oxygen-consumption activity was assessed using an oxygen-sensitive fluorogenic probe and measured with a microplate reader.

To functionally characterize mitochondrial respiration, sequential treatments with oligomycin (Sigma, O4876-5MG) (ATP synthase inhibitor), FCCP (Sigma, C2920-10MG) (mitochondrial uncoupler), and rotenone (Sigma, R8875-1G) (complex I/III inhibitors) were performed to evaluate basal respiration, maximal respiratory capacity, and spare respiratory capacity. Fluorescence values were normalized to cell number and expressed as relative OCR activity.

#### Mitochondrial membrane potential (ΔΨm)

Cells were incubated with JC-1 dye (2  µM; Dojindo, MT09-10) for 30  min at 37 °C in complete medium. For JC-1 validation, cells were treated with the uncoupler carbonyl cyanide m-chlorophenyl hydrazone (CCCP, 10 μM, 30 min) (Sigma, C2759-100MG) to confirm complete mitochondrial depolarization. Immediately afterwards, ΔΨm was quantified by flow cytometry on a BD Accuri C6 Plus, recording FL1 (530/30 nm) and FL2 (585/40 nm) signals from 1 × 10⁴ events.

#### Mitophagy detection

Mitophagy was assessed using the Mitophagy Detection Kit (Dojindo, MD01-10), which measures the colocalization of mitochondria and lysosomes via pH-dependent fluorescence shift. Quantification was performed by flow cytometry on a BD Accuri C6 Plus on ≥10,000 live events per sample. As a positive control, cells were treated with the mitochondrial uncoupler CCCP (10 μM, 30 min), which induced a robust increase in mitophagy signal and validated the assay specificity.

#### Western blotting

Proteins (20–30 µg) were separated on 10% SDS–PAGE gels, transferred to PVDF (Millipore), and probed with antibodies against MFN1, MFN2, DRP1, FIS1, COX-I, SDH-A, PINK1, Ub-S65, PGC1α, NRF1, NRF2, H4, GAPDH and β-actin (Supplementary Table 1). Immunoreactive signals were detected with a horseradish peroxidase-conjugated secondary antibody (ImmunoReagents) and reacted with ECL plus reagent (Merck Millipore). All the antibodies were used according to the manufacturer's instructions. All information regarding antibodies and dilutions is reported in Supplementary Table 1. The membranes were analysed using Quantity One 1-D analysis software (Bio-Rad). Normalization was performed using β-actin (ACTB), GAPDH, or H4 as internal controls.

#### Immunocytochemistry

We grew cells on cover slides in MW24 plates and then fixed them in 4% formaldehyde solution (Sigma) for 15 min at room temperature. The cells were washed three times with PBS and permeabilized with 0.3% Triton X-100 (Sigma, X100-5ML) in PBS for 15 min. Subsequently, after being washed with PBS, the cells were incubated for 1 h at room temperature in a blocking solution composed of 0.1% Triton X-100 and 5% of bovine serum in PBS. The cells were incubated with primary antibody in blocking overnight at 4 °C. All the antibodies were used according to the manufacturer's instructions. All information regarding antibodies and dilutions (company and catalogue numbers) is reported in Supplementary Table 1. The following day the cells, after three washes in PBS of 5 min each, were incubated for 1 h at room temperature in the dark with secondary antibodies conjugated to fluorophores. Then three washes with PBS for 5 min each were carried out and the slides were mounted. Nuclear staining was performed by a DAPI mounting medium (ABCAM) and micrographs were taken under a fluorescence microscope (Zeiss). The percentage of positive cells was calculated by counting at least 500 cells in different microscope fields. For imaging-based validation of mitophagy, colocalization between LAMP1 and MitoTracker signals was analyzed in ImageJ/Fiji using the Coloc2 plugin. Quantification was performed by calculating Manders' coefficients, representing the fraction of MitoTracker signal overlapping with LAMP1, on ≥20 randomly selected fields.

#### 8-oxo-dG immunofluorescence

Oxidative DNA damage was quantified by immunostaining for 8-oxo-2′-deoxyguanosine (8-oxo-dG) essentially as described by Ohno [35], with minor adaptations for MUSE, MSC, and Non-MUSE cultures. Briefly, cells (2 × 10^5^) were plated on poly-L-lysine-coated coverslips, exposed to 0.3 mM H₂O₂, and fixed in ice-cold methanol (–20 °C, 10 min). To selectively visualize mitochondrial or nuclear 8-oxo-dG signal, different DNA denaturation steps were applied: for mitochondrial DNA, slides were immersed in 50 mM NaOH/50% ethanol for 7 min at room temperature and then rinsed three times in PBS (3 min each); for nuclear DNA, slides were treated with 2 N HCl for 10 min at room temperature and rinsed thoroughly in PBS. Following DNA denaturation, residual RNA was digested using RNase A (5 mg/ml, 1 h, 37 °C), and slides were blocked with BlockAce (10 min). 8-oxo-dG was detected using anti-8-oxo-dG mouse monoclonal antibody (Santa Cruz, SC-130914; 1:100, 2 h, RT, humidified dark chamber), followed by Alexa Fluor 488-conjugated anti-mouse IgG secondary antibody (1:300, 40 min). Nuclei were counterstained with DAPI.

### Statistical analysis

Data are presented as mean ± SD from *n* ≥ 3 independent experiments. Significance was assessed by one-way ANOVA with Tukey's post-hoc test (GraphPad Prism 10). Differences were considered significant at *p* < 0.05. Time-course experiments were analyzed using two-way ANOVA considering time and cell treatment as independent variables, followed by Šídák's multiple-comparison post hoc test. Data normality was assessed using the Shapiro–Wilk test prior to parametric analysis.

## Results

### MUSE cells preserve mitochondrial DNA integrity and activate efficient DNA repair mechanisms under oxidative stress

MUSE cells were first isolated from commercial human MSC preparations by magnetic sorting for the pluripotency-associated antigen SSEA-3. To obtain a more homogeneous and stem-like population, the SSEA-3⁺ fraction was cultured for 7 days in suspension, allowing cells to form non-adherent spheroids that enrich for the MUSE phenotype. Spheroids were then dissociated and replated on tissue-culture plastic; after 24 h of adhesion, the cells were ready for experimentation [36–38]. The efficiency of the purification was assessed by flow cytometry: MUSE cultures showed >91% positivity for SSEA-3, whereas both MSCs and Non-MUSE fractions remained below 3% (Sup. Figure S1). Stemness was further validated by the expression of pluripotency markers OCT3/4, SOX2, and NANOG, consistent with previous reports (data not shown). Once purity was confirmed, MUSE cells, parental MSCs and SSEA-3-negative Non-MUSE fractions were all exposed to a single oxidative pulse (300 µM H₂O₂ for 30 min) designed to mimic a transient burst of niche ROS [39]. Continuous DCF-DA recordings over 48 h revealed a clear divergence in redox kinetics. The fluorescence trajectory in MUSE cells climbed more slowly and plateaued at ~15% lower than peroxide-treated MSCs and ~25% lower than peroxide-treated Non-MUSE cells throughout the 6- to 48-h window (Figure 1A). Even under basal conditions, spontaneous ROS accumulation remained the lowest in MUSE cultures, ending ~20% below untreated MSCs and ~25% below untreated Non-MUSE cells at 48 h. A gradual increase in DCF-DA fluorescence was also detected in untreated cultures during the 48-h acquisition period. This phenomenon likely reflects cumulative probe oxidation during prolonged live monitoring together with physiological metabolic ROS production and progressive cell growth, rather than an acute oxidative insult.

**Figure 1.**
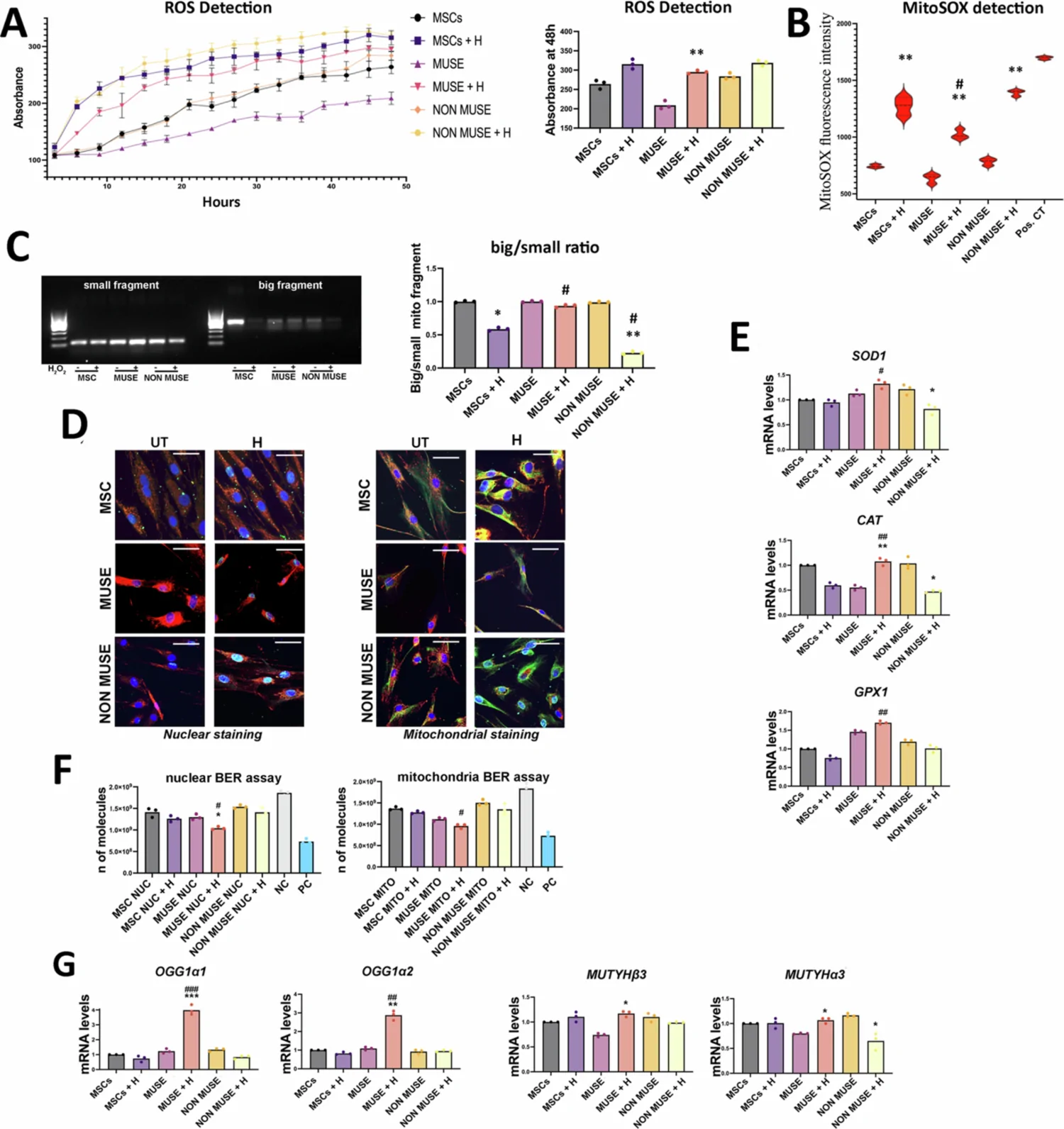
MUSE cells limit mitochondrial ROS accumulation and preserve mitochondrial genome integrity through early antioxidant and BER activation (A) ROS levels were measured in real time using DCF-DA fluorescence over 48 h following exposure to 300 µM H₂O₂. (B) Mitochondrial superoxide accumulation measured by MitoSOX-based fluorometric assay 1 h after H₂O₂ exposure. (C) Long-range PCR of mitochondrial DNA (mtDNA) performed 24 h post-stress, amplifying small (221 bp) and large (8.9 kb) fragments. The histogram shows the large-to-small fragment ratio, which serves as an indicator of mtDNA integrity. (D) Representative immunofluorescence images showing 8-oxo-dG accumulation in nuclei (left) and mitochondria (right) at 24 h post H₂O₂ (H) (20X magnification). The 8-oxo-dG is shown in green; mitochondria are stained with MitoTracker Red. Quantification was performed by analysing at least 500 cells per condition. Scale bar 100 µm. (E) Relative mRNA levels of antioxidant genes (*SOD1, CAT, GPX1*) 1 h post-stress. (F) Base Excision Repair (BER) activity measured in mitochondrial (top) and nuclear (bottom) extracts. (G) qPCR analysis of *OGG1* and *MUTYH* isoforms 1 h after stress exposure. The mRNA levels were normalized to GAPDH mRNA expression, which was selected as an internal control. All data are presented as mean ± SD from n = 3 independent experiments. Statistical significance: *p* < 0.05 (*), *p* < 0.01 (**), *p* < 0.001 (***) for treated vs. untreated within the same population; #*p* < 0.05 (#), *p* < 0.01 (##), *p* < 0.001 (###) for comparison vs MSC + H₂O₂.

To determine whether exogenous H₂O₂ also induced mitochondrial ROS accumulation, mitochondrial superoxide levels were quantified using a MitoSOX-based fluorometric assay. Following oxidative stress exposure, MSCs and Non-MUSE cells exhibited a marked increase in mitochondrial superoxide levels, whereas MUSE cells displayed significantly lower mitochondrial ROS accumulation (Figure 1B).

One of the main detrimental effects of ROS is damage to cellular DNA. Mitochondrial DNA is particularly susceptible to ROS due to its proximity to the electron transport chain, the primary source of intracellular ROS, and the absence of protective nucleosomes, which shield nuclear DNA [40–42]. We therefore probed mitochondrial genome stability. A long-range PCR assay targeting large (8.9 kb) and small (221 bp) mitochondrial DNA fragments revealed a marked preservation of mtDNA integrity in MUSE cells 24 h after stress induction, as indicated by the maintenance of the large-to-small fragment ratio (Figure 1C). In contrast, both MSCs and Non-MUSE cells exhibited a pronounced reduction in this ratio following H₂O₂ treatment, suggestive of extensive mitochondrial DNA fragmentation [41] (Figure 1C). The molecular nature of this protection was confirmed by immunofluorescence for 8-oxo-2′-deoxyguanosine: at 24 h, MUSE nuclei and mitochondria accumulated significantly fewer 8-oxo-dG adducts than MSCs or Non-MUSE cells. Notably, the few oxidative lesions observed in MUSE cells at 1 h were effectively resolved by 24 h, indicating prompt activation of DNA repair mechanisms [43] (Figure 1D).

To determine whether the lower lesion burden in MUSE cells reflected an early antioxidant response, we quantified transcript levels of catalase (*CAT*), superoxide dismutase (*SOD1*), and glutathione peroxidase (*GPX1*) transcripts one hour after the oxidative pulse [44–46]. At baseline, *SOD1* and *GPX1*were already elevated in MUSE cells compared to MSCs and Non-MUSE cells, while *CAT* levels were slightly lower. 1 hour following H₂O₂ treatment, MUSE cells exhibited a robust transcriptional upregulation of all three genes: *CAT* increased by ~60%, *SOD1* by ~35%, and *GPX1* by ~25% relative to their own untreated controls. In contrast, MSCs showed only a mild reduction of *CAT* and negligible changes in *SOD1* and *GPX1*, while Non-MUSE cells failed to induce any of the antioxidant genes and, in fact, displayed a slight decrease in *SOD1* and *CAT* expression. When comparing the three populations after H₂O₂ exposure, MUSE cells displayed the highest absolute expression levels for all three antioxidant genes, significantly surpassing both MSCs and Non-MUSE cells (Figure 1E).

The above data suggest that MUSE cells quench ROS more efficiently than MSCs. However, even a transient ROS burst, if successfully neutralized, may still inflict damage on macromolecules. We therefore investigated whether this early transcriptional response was complemented by a more effective mitochondrial DNA repair mechanism in MUSE cells. Base Excision Repair (BER) activity, the primary pathway for repairing oxidative lesions in mtDNA [46,47], was assessed in mitochondrial extracts isolated from each cell population. Mitochondria and nuclei purification was validated *via* western blot analysis using mitochondrial and nuclear markers (Sup. Figure 2). Enzymatic assays revealed a marked increase in BER activity in MUSE cells, particularly 24 h post-H₂O₂ exposure, in both mitochondrial and nuclear compartments, while MSCs exhibited only a marginal gain, and Non-MUSE cells remained unchanged (Figure 1F). Finally, we examined whether the superior BER activity in MUSE cells is underpinned by early transcriptional up-regulation of the two major 8-oxo-dG glycosylases, OGG1 and MUTYH, whose splice variants localise either to the nucleus (*OGG1α1, MUTYHß3*) or to mitochondria (*OGG1α2, MUTYHα3*) [48–50]. RT-qPCR carried out 1 h after the oxidative pulse revealed a sharp, isoform-selective response in MUSE cells (Figure 1G). The nuclear *OGG1α1* transcript in MUSE cells after stress increased nearly four-fold, and its mitochondrial counterpart *OGG1α2* rose ~three-fold relative to untreated MUSE controls. The *MUTYH* isoforms showed milder increments of ~25–30% (ß3 and α3, respectively). In MSCs, the only significant change was a slight elevation of *MUTYHß3*, while all other isoforms remained at baseline. Non-MUSE cells failed to up-regulate any glycosylase and, in the case of the mitochondrial *MUTYHα3*, even showed a small decline. Thus, MUSE cells reinforce their DNA repair response through a rapid, dual-compartment boost of *OGG1*, complemented by a moderate rise in *MUTYH*, a concerted transcriptional pattern that is absent in the comparator populations and likely critical for their enhanced mtDNA protection. BER activity indicates a higher general BER capacity toward a defined uracil-containing substrate, whereas OGG1/MUTYH expression and 8-oxo-dG staining support activation of oxidative-lesion repair pathways.

**Figure 2.**
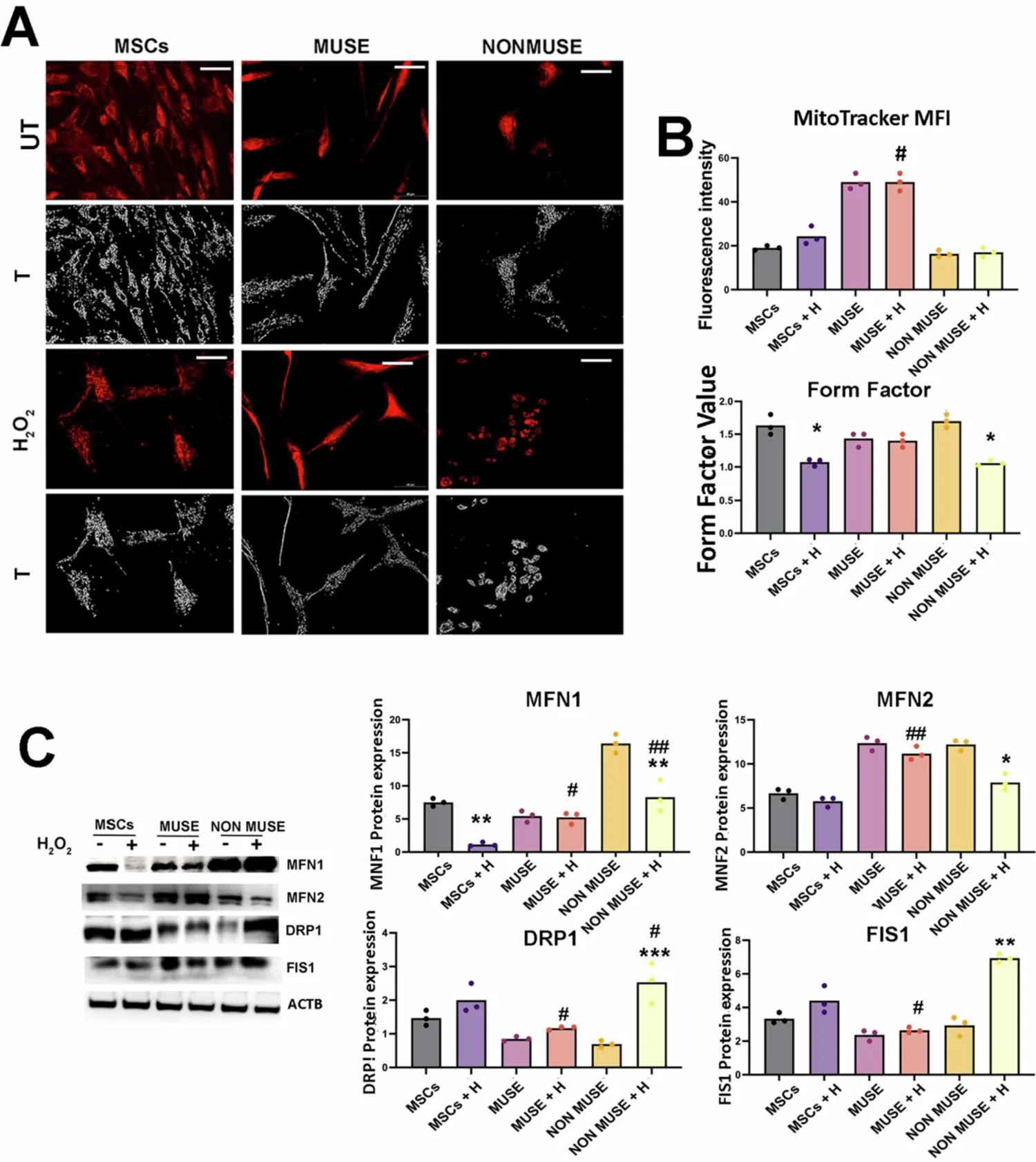
MUSE cells maintain mitochondrial morphology under oxidative stress. (A) Representative images of mitochondria stained with MitoTracker Red in MSCs, MUSE, and Non-MUSE cells at baseline and 48 h after 300 μM H₂O₂ exposure. Images were acquired at 20X magnification. For each condition, the applied threshold (T) used for morphometric analysis with Mitochondria Analyzer software is shown. Thresholding was applied exclusively for image segmentation prior to automated morphometric analysis. Scale bar 100 µm (B) Quantification of mitochondrial mean fluorescence intensity (MFI, top panel) and Form Factor (bottom panel). MFI analysis was performed on at least 500 cells per condition, whereas Form Factor quantification was performed on at least 500 cells per condition. (C) Western blot analysis of mitochondrial fusion (MFN1, MFN2) and fission (DRP1, FIS1) proteins in the three populations ± H₂O₂. Bar graphs show densitometric quantification of protein levels normalized to ACTB, which was used as loading control. All data are presented as mean ± SD (n = 3 from independent experiments). *p < 0.05, **p < 0.01, ***p < 0.001 vs. respective untreated controls; #*p* < 0.05, ##*p* < 0.01 vs. MSCs + H₂O₂.

Taken together, the temporal cascade, rapid ROS dampening, preservation of long mtDNA amplicons, reduced 8-oxo-dG accumulation, early antioxidant gene up-shift, and a selective surge in mitochondrial BER enzymes, demonstrate that MUSE cells counter an acute oxidative challenge through a tightly integrated programme that couples reactive-oxygen clearance with proactive mitochondrial genome repair.

### MUSE cells maintain mitochondrial morphology following oxidative stress

ROS can profoundly impact mitochondrial functions, including their biogenesis, fusion, fission, and clearance. Our primary aim was to evaluate mitochondrial shape dynamics and network organization. To evaluate mitochondrial content, we quantified the mean fluorescence intensity (MFI) of MitoTracker Red signal (Figure 2A). At baseline, MUSE cells displayed significantly higher MitoTracker fluorescence compared to MSCs (~20% increase) and Non-MUSE cells (~25% increase), indicating a greater mitochondrial mass per cell. Following exposure to 300 µM H₂O₂, fluorescence intensity remained largely unchanged in all populations (Figure 2B). We therefore next asked whether this phenomenon was accompanied by changes in mitochondrial morphology and performance [51].

To evaluate the impact of oxidative stress on mitochondrial structure, we assessed mitochondrial morphology using two complementary approaches: Mitotracker-based fluorescence imaging combined with morphometric analysis *via* the Mitochondria Analyzer software [33], and western blotting of key regulators of mitochondrial fission and fusion.

Quantitative morphometric analysis revealed that MUSE cells maintained an elongated mitochondrial network 48 h after H₂O₂ exposure, with a form factor (FF) value consistently around 1.5, comparable to untreated controls. The form factor is a dimensionless metric calculated as (perimeter^2^)/(4π × area) [52–54], which reflects both the elongation and complexity of mitochondrial structures: higher values indicate more tubular and interconnected mitochondria, while lower values suggest fragmentation and sphericity. As such, FF provides a robust quantitative descriptor of mitochondrial morphology, especially under stress conditions where fission–fusion dynamics are disrupted. In contrast to MUSE cells, both MSCs and Non-MUSE cells showed a pronounced morphological shift toward fragmented, rounded mitochondria following oxidative challenge, with FF values decreasing to approximately 1.0, indicative of mitochondrial fission and structural compromise (Figure 2B).

These observations were corroborated by Western blot analysis of mitochondrial dynamics-related proteins. In MSCs and Non-MUSE cells, oxidative stress triggered a substantial downregulation of mitochondrial fusion proteins mitofusin-1 (MFN1) and mitofusin-2 (MFN2), along with a concomitant upregulation of fission-related proteins dynamin-related protein 1 (DRP1) and fission 1 (FIS1) [55–57]. Conversely, MUSE cells exhibited no significant alterations in the expression of these proteins following H₂O₂ treatment, suggesting a stable balance between fusion and fission processes (Figure 2C).

### MUSE cells preserve mitochondrial respiratory function following oxidative stress

To determine whether preservation of mitochondrial morphology translated into maintained mitochondrial function, mitochondrial respiratory activity was next evaluated using a fluorescence-based oxygen-consumption assay. Following H₂O₂ exposure, MSCs and Non-MUSE cells exhibited a marked reduction in mitochondrial respiratory activity, characterized by decreased maximal respiration and spare respiratory capacity. In contrast, MUSE cells maintained significantly higher respiratory performance under oxidative stress conditions (Figure 3A), indicating improved preservation of mitochondrial bioenergetic fitness.

**Figure 3.**
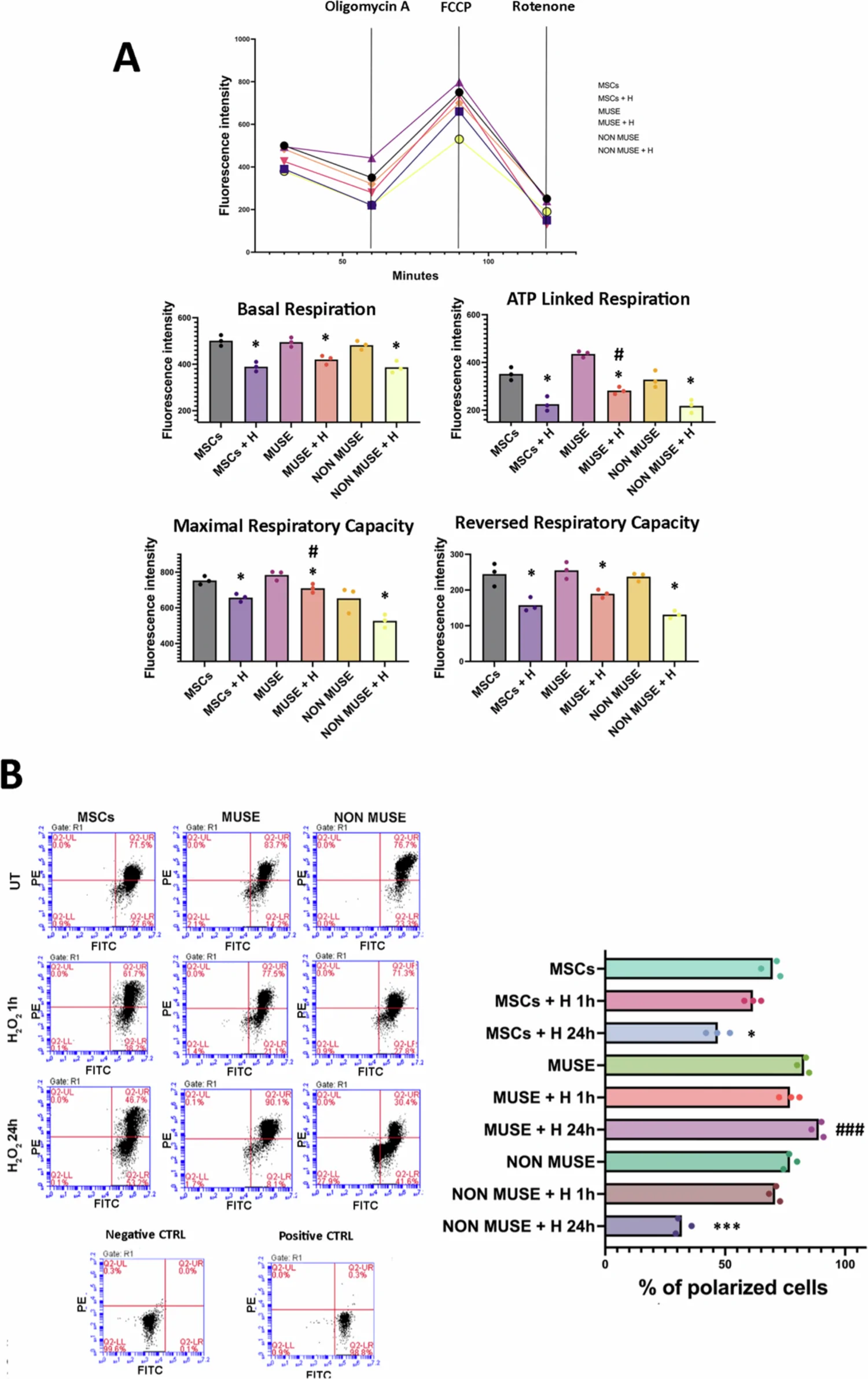
MUSE cells preserve mitochondrial respiratory function and membrane potential under oxidative stress (A) Mitochondrial respiratory activity was evaluated 24 h after H₂O₂ exposure using a fluorescence-based oxygen-consumption assay. OCR profiles were obtained following sequential treatment with oligomycin, FCCP, and rotenone. Histograms summarize basal respiration, ATP-linked respiration, maximal respiration, and spare respiratory capacity. (B) Mitochondrial membrane potential (ΔΨm) was assessed by flow cytometry at baseline, 1 h, and 24 h after oxidative stress. The bar graph summarizes the percentage of cells with polarized mitochondria. As controls, CCCP-treated cells were used as the positive control (loss of polarization), whereas unstained cells served as the negative control. All data are presented as mean ± SD (n = 3 from independent experiments). *p < 0.05, **p < 0.01, ***p < 0.001 vs. respective untreated controls; ###p < 0.001 vs. MSCs + H₂O₂.

Finally, we assessed mitochondrial functional integrity by measuring the membrane potential (ΔΨm) with JC-1. This cationic dye accumulates in mitochondria in a potential-dependent manner, forming red fluorescent aggregates in polarized mitochondria and green monomers in depolarized mitochondria [58]. In basal condition, cells with red-aggregate were 84% ± 5% in MUSE sample, 72% ± 3% in MSCs and 77% ± 4% in Non-MUSE cells. One hour after stress, the polarised fraction decreased modestly in all populations. By 24  h, MUSE cells had already recovered baseline ΔΨm, while MSCs and Non-MUSE cells remained markedly depolarised (Figure 3B). These complementary data confirm that MUSE mitochondria are intrinsically more resistant to stress-induced depolarisation and can rapidly re-establish membrane potential, underscoring their superior bioenergetic stability under oxidative challenge.

### MUSE cells display enhanced mitophagy activation in response to oxidative stress

Given that MUSE cells preserved mitochondrial morphology and maintained membrane potential under oxidative stress, we next sought to investigate whether this stability reflected more effective mitochondrial quality control mechanisms. In particular, we assessed the activation of mitophagy, the selective degradation of damaged mitochondria, as a key arm of the mitochondrial surveillance system. To this end, we employed three complementary approaches: (i) immunocytochemistry combining lysosomal (LAMP1) and mitochondrial (Mitotracker) markers to visualize mitophagic events, (ii) quantitative analysis using a commercial Mitophagy Detection Kit, and (iii) western blotting for critical regulators of mitophagy, including phosphorylated ubiquitin at serine 65 (Ub-S65) and PTEN-induced kinase 1 (PINK1) [59,60].

Immunocytochemical analysis revealed a substantial increase in colocalization events between LAMP1 and Mitotracker signals in MUSE cells 48 h after H₂O₂ treatment, visualized as distinct overlapping puncta, indicating the engulfment of mitochondria by lysosomes (Figure 4A). Quantitative colocalization analysis using Manders' coefficients confirmed this observation: the fraction of Mitotracker signal overlapping with LAMP1 was higher in MUSE cells compared to both MSCs and Non-MUSE cells. While MSCs displayed only a modest increase in colocalization and Non-MUSE cells remained largely unchanged, MUSE cells exhibited a more than two-fold rise in LAMP1–Mitotracker overlap following oxidative stress, indicating a robust induction of mitophagy. This was paralleled by results from the Mitophagy Detection Kit, which also showed a significant increase in dual-labelled mitochondria–lysosome puncta in MUSE cells following oxidative stress compared to the other populations (Figure 4B).

**Figure 4.**
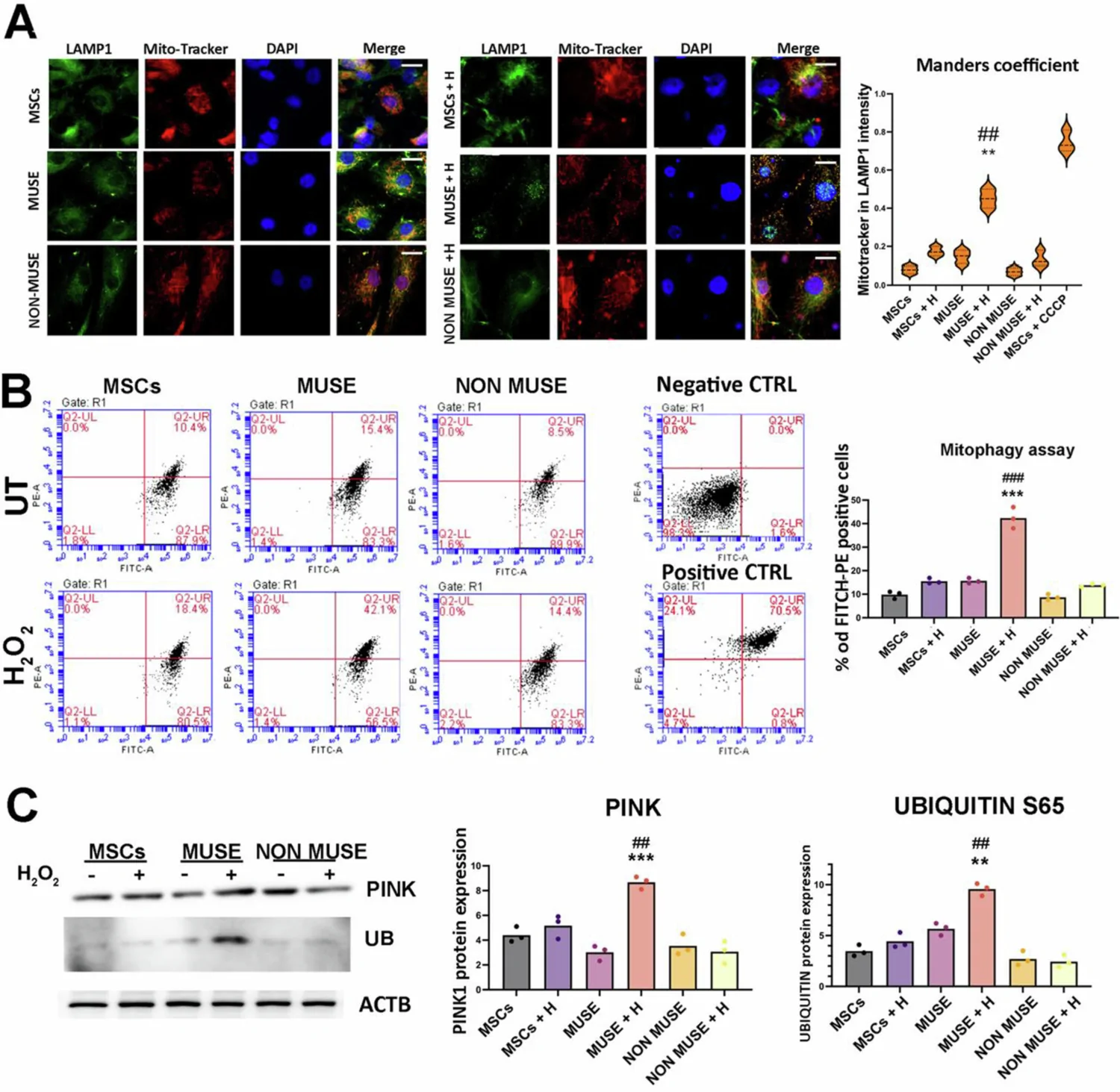
MUSE cells activate PINK1-dependent mitophagy to clear damaged mitochondria. A) Representative immunofluorescence images showing the co-localization between lysosomes (LAMP1, green) and mitochondria (MitoTracker, red), with nuclei counterstained with DAPI (blue), in basal conditions and 48 h after 300 μM H₂O₂ exposure (20X magnification). Scale bar 50 µm. The violin plot represents quantification of colocalization using Manders’ coefficient (fraction of MitoTracker signal overlapping with LAMP1), analyzed with the Coloc2 plugin in ImageJ. Positive control was obtained with CCCP, and negative control with bafilomycin A1. Quantification was performed on at least 30 randomly selected fields per condition (B) Representative flow-cytometry dot plots from the Mitophagy Detection Kit and corresponding quantification histogram showing the percentage of cells positive for dual mitochondrial and lysosomal signals. As controls, CCCP was used as a positive inducer of mitophagy, whereas the negative control corresponds to unstained cells. For each condition, at least 10,000 live-cell events were acquired. (C) Western blot analysis of phosphorylated ubiquitin at Ser65 (Ub-S65) and PINK1 in the three populations ± H₂O₂. Densitometric quantifications (adjacent histograms) were normalized to ACTB, which served as the loading control. All data are presented as mean ± SD (n = 3 from independent experiments). **p < 0.01, ***p < 0.001 vs. respective untreated controls; ##*p* < 0.01, ###*p* < 0.001 vs. MSCs + H₂O₂.

Western blot analysis further supported these results and identified the molecular pathway driving mitophagy activation. MUSE cells showed a clear upregulation of phospho-Ub-S65 and PINK1 after H₂O₂ exposure, suggesting active recruitment of the PINK1/Parkin-mediated mitophagy pathway [61].

(Figure 4C). Although MSCs exhibited a modest increase in mitophagy markers following stress, the magnitude of activation was notably lower than in MUSE cells. Non-MUSE cells, by contrast, displayed only a weak or delayed mitophagic response under the same conditions.

These data highlight the superior capacity of MUSE cells to promptly initiate mitophagy upon oxidative injury, thereby facilitating the removal of dysfunctional mitochondria.

### Enhanced mitobiogenesis and mitochondrial regeneration in MUSE cells following oxidative stress

Finally, to investigate mitochondrial biogenesis in response to oxidative stress, we evaluated the expression of mitochondrial- and nuclear-encoded respiratory chain subunits *via* western blot using a dual-target antibody cocktail. This included subunit I of Complex IV (COX-I), encoded by mitochondrial DNA (mtDNA), and subunit A of Complex II (SDH-A), encoded by nuclear DNA (nDNA).

Under basal conditions, MUSE cells displayed a higher COX-I/SDH-A ratio compared to MSCs and Non-MUSE cells, indicating a more active mitobiogenic state. Forty-eight hours after the 300 µM H₂O₂ pulse, this ratio significantly increased in MUSE cells, consistent with enhanced mitochondrial biogenesis. In contrast, both MSCs and Non-MUSE cells showed a reduction in COX-I/SDH-A ratio post-stress, suggesting an impaired capacity for mitochondrial regeneration (Figure 5A).

**Figure 5.**
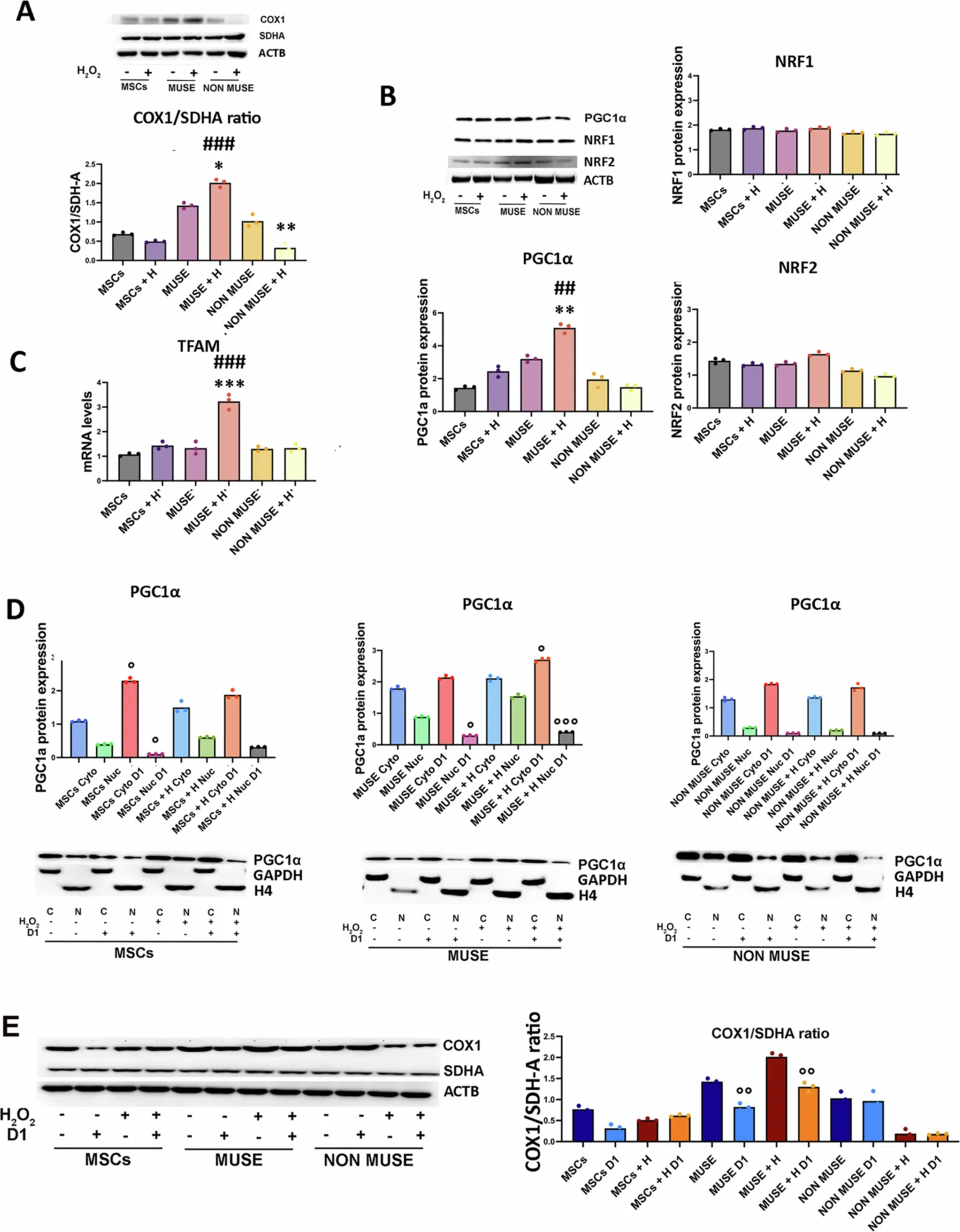
Enhanced mitochondrial biogenesis in MUSE cells via PGC1α–NRF2 axis. (A) Western blot analysis of mitochondrial (COX-I) and nuclear-encoded (SDHA) respiratory chain subunits under basal conditions and 48 h after H₂O₂ treatment. Densitometric quantifications were normalized to ACTB, which served as the loading control. COX-I/SDHA ratio, shown in the graph, was used as an index of mitochondrial biogenesis. (B) Expression levels of mitochondrial biogenesis regulators PGC1α, NRF1, and NRF2, which were evaluated by western blot before and after oxidative stress. The densitometric quantification of protein levels normalized with ACTB, which was used as loading control. (C) TFAM mRNA expression was quantified by qPCR 48 h after H₂O₂ exposure. The mRNA levels were normalized to GAPDH mRNA expression, which was selected as an internal control. (D) Cytoplasmic (labelled as C) and nuclear (labelled as N) protein levels of PGC1α, evaluated by western blot before and after oxidative stress either in presence or absence of the PGC1α inhibitor SR-18292 (labelled as D1). Densitometric quantifications were normalized to GAPDH for the cytoplasmic fraction and to histone H4 for the nuclear fraction. (E) Western blot analysis of mitochondrial (COX-I) and nuclear-encoded (SDHA) respiratory chain subunits under basal conditions and after H₂O₂ treatment either in presence or absence of the PGC1α inhibitor SR-18292 (D1). Densitometric quantifications were normalized to ACTB, which served as the loading control. COX-I/SDHA ratio, shown in the graph, was used as an index of mitochondrial biogenesis. All data are presented as mean ± SD (n = 3 from independent experiments). **p < 0.01, ***p < 0.001 vs. respective untreated control; ##*p* < 0.01, ###*p* < 0.001 vs. MSCs + H₂O₂; °*p* < 0.05, °°*p* < 0.01, °°°*p* < 0.001 vs. corresponding no drug-treated condition.

To gain further insight into the transcriptional mechanisms underlying the enhanced mitobiogenesis observed in MUSE cells, we analyzed the expression levels of the key mitochondrial biogenesis regulators PGC-1α, NRF1, and NRF2 [62]. As shown in Figure 5B, PGC1α expression was markedly upregulated in MUSE cells 48 h after oxidative stress, while only modest changes were detected in MSCs. Interestingly, NRF1 expression remained relatively stable across all cell populations, indicating that this factor may not be differentially regulated at the protein level in response to oxidative stress. In contrast, though total NRF2 protein levels showed only modest changes following oxidative stress, subcellular fractionation experiments revealed enhanced nuclear accumulation of NRF2 selectively in MUSE cells, supporting activation of NRF2-dependent transcriptional programmes (Sup. Figure 3).

The increased levels of PGC1α and NRF2 may suggest that these transcription factors may upregulate the mRNA expression of TFAM, a key downstream effector of mitochondrial biogenesis and mitochondrial DNA replication [63–65]. As depicted in Figure 5C, MUSE cells exhibited a significant elevation in TFAM mRNA levels following H₂O₂ treatment, whereas MSCs and Non-MUSE cells showed minimal or no induction.

To further validate the involvement of PGC-1α in the regulation of mitochondrial biogenesis, and specifically its nuclear translocation, we pharmacologically inhibited the pathway using selective compounds SR-18292 (D1) for PGC1α and ML385 for NRF2. Cells were pretreated overnight with the inhibitors, followed by H₂O₂ exposure. Nuclear and cytoplasmic protein fractions were collected 4 hours after stress induction to assess the intracellular distribution of these transcriptional regulators. Under stress conditions, MUSE cells exhibited a marked translocation of PGC1α from the cytoplasm to the nucleus, a critical step for initiating the transcriptional cascade governing mitochondrial biogenesis [66]. This nuclear accumulation was significantly more robust in MUSE cells compared to MSCs and Non-MUSE cells, suggesting a stronger and more efficient engagement of the mitochondrial regeneration programme (Figure 5D). Upon PGC-1α inhibition, a substantial decrease in its nuclear localization was observed in all three cell populations. However, the effect was most pronounced in MUSE cells, in which nuclear PGC-1α levels were sharply reduced. This finding underscores the central reliance of MUSE cells on the nuclear activity of PGC-1α to sustain their biogenic response under oxidative conditions (Figure 5D). In parallel fractionation experiments, NRF2 inhibition reduced nuclear NRF2 accumulation by ~50% only in MUSE cells, whereas NRF2 localisation in MSCs and Non-MUSE cells remained essentially unchanged (data not shown). PGC1-*α* inhibition selectively impaired mitochondrial biogenesis in MUSE cells, as indicated by a marked reduction in the COX1/SDHA ratio following combined H₂O₂ exposure and PGC1α inhibitor treatment (Figure 5E). In contrast, MSCs and Non-MUSE cells showed no further decrease in COX1 expression compared to H₂O₂ alone. Importantly, pharmacological inhibition of PGC-1α not only reduced its nuclear accumulation but also selectively impaired mitochondrial biogenesis in MUSE cells, indicating that PGC1α activity is functionally required to sustain mitochondrial regeneration under oxidative stress.

## Discussion

Reactive oxygen species (ROS) are ubiquitous byproducts of cellular metabolism and signalling, but their accumulation imposes a major threat to tissue homeostasis. Adult stem cells, which are responsible for tissue maintenance, repair, and regeneration, are particularly sensitive to oxidative stress: moderate ROS levels can act as signalling cues for activation and differentiation, but excessive or sustained ROS exposure damages DNA, impairs mitochondrial function, and depletes the regenerative pool. Understanding how different stem cell populations sense, buffer, and adapt to oxidative stress is therefore critical for advancing both fundamental stem cell biology and regenerative medicine.

Mitochondria lie at the heart of every decision a stem cell makes, from whether to remain quiescent or to embark on a programme of proliferation and differentiation, to whether it survives or succumbs to a sudden burst of oxidative stress [67]. Our data place human MUSE stem cells at the extreme end of this metabolic spectrum. Confronted with an acute pulse of hydrogen peroxide, MUSE cells execute an unusually rapid mitochondrial quality-control sequence that couples PINK1-dependent mitophagy to an equally prompt wave of PGC-1α–driven biogenesis [68,69]. The net result is a seamless ‘remove-and-replace’ cycle initiated within the first hours following oxidative stress. By compressing the vulnerable interval during which damaged organelles are cleared but not yet replaced, MUSE cells preserve membrane potential, preserve mitochondrial bioenergetic competence and avoid the apoptotic drift that undermines conventional MSCs and Non-MUSE stromal cells [20]. These observations are consistent with our earlier demonstration that MUSE cells resist apoptotic execution under stress, and provide a mechanistic explanation for that phenotype: by maintaining mitochondrial integrity through coordinated quality control, MUSE cells prevent the cytochrome c release and caspase cascade activation that would otherwise commit them to programmed cell death. This positions mitochondrial quality control as an upstream determinant of the apoptotic threshold in stem cell populations.

While MSCs comprise a heterogeneous pool of cells with varying degrees of ROS resistance and mitochondrial quality control (MQC) capacity, allowing them to mount a broad, albeit less specialized, stress response, MUSE stem cells emerge as a distinct subset with enhanced mitochondrial plasticity and stress adaptability within the broader MSC population. As a rare but indispensable subset of MSCs, MUSE cells appear to function as a highly stress-resilient subpopulation, executing rapid stress-adaptive responses to maintain tissue homoeostasis under oxidative duress. This hierarchical organization, where MUSE stem cells act as precision responders, supported by the MSC ‘safety net’, may reflect an evolutionary adaptation to balance rapid damage control with broader tissue surveillance.

MUSE cells display a remarkable ability to survive and maintain their function in hostile environments, a trait that distinguishes them from conventional MSCs and Non-MUSE stromal cells. This property makes them highly attractive for regenerative medicine, particularly in pathological contexts such as inflammation, ischaemia, and tissue injury, conditions often characterized by elevated oxidative stress [70].

Delving into the details of how MUSE stem cells respond to oxidative stress, we observed that these cells maintain mitochondrial DNA integrity and morphology following H₂O₂ exposure. Although exogenous H₂O₂ does not represent a mitochondria-specific insult, extracellular H₂O₂ can rapidly diffuse across cellular compartments, including mitochondria, where it may promote secondary mitochondrial ROS amplification through ROS-induced ROS-generation mechanisms [71]. Consistent with this concept, we observed increased mitochondrial superoxide accumulation following H₂O₂ exposure, particularly in MSCs and Non-MUSE cells, whereas MUSE cells displayed significantly lower mitochondrial ROS levels under the same conditions. These findings support the involvement of mitochondrial oxidative stress in our experimental model and further suggest that MUSE cells possess enhanced mechanisms to preserve mitochondrial redox homoeostasis under oxidative stress conditions. Unlike MSCs and Non-MUSE cells, MUSE cells retained elongated mitochondrial structures and maintained mtDNA integrity, as evidenced by the large-to-small fragment PCR ratio and reduced levels of 8-oxo-dG [72]. These results suggest a highly effective mechanism in MUSE cells for preventing and repairing oxidative damage to mitochondrial DNA, likely via enhanced base excision repair (BER) activity [73]. The BER assay employed in this study was designed to evaluate general BER-associated repair capacity using a uracil-containing DNA substrate and does not directly measure enzymatic excision of oxidative lesions such as 8-oxo-dG. Nevertheless, the concomitant reduction in 8-oxo-dG accumulation together with increased OGG1 and MUTYH expression supports activation of oxidative DNA-damage repair pathways in MUSE cells under oxidative stress conditions.

MUSE cells maintained significantly higher ΔΨm values after oxidative insult, indicating superior capacity to preserve electrochemical gradient and mitochondrial bioenergetic competence. The preservation of mitochondrial respiratory activity observed in MUSE cells indicates that the mitochondrial adaptive responses identified in this study are not limited to structural preservation but are functionally associated with maintenance of mitochondrial bioenergetic competence under oxidative stress conditions.

A key aspect of mitochondrial quality control is the dynamic balance between mitophagy and biogenesis [74]. We demonstrated that MUSE cells activate mitophagy robustly in response to oxidative stress, as shown by increased colocalization of mitochondrial and lysosomal markers, elevated mitophagy kit signal, and upregulation of PINK1 and phosphorylated ubiquitin (Ub-S65) [75,76]. This response likely enables MUSE cells to rapidly eliminate dysfunctional mitochondria and avoid secondary ROS accumulation or apoptotic signalling [77,78].

Complementarily, MUSE cells also displayed a strong induction of mitochondrial biogenesis, evidenced by elevated COX-I/SDH-A protein ratios and increased expression of transcriptional regulators [79]. Notably, MUSE cells exhibited higher basal mitobiogenesis, and this capacity was further augmented following stress, unlike MSCs and Non-MUSE cells, which exhibited a stress-induced suppression of mitochondrial biogenesis. We focused on the roles of PGC1α and NRF2, two transcriptional regulators known to orchestrate mitochondrial and antioxidant responses. MUSE cells showed significant upregulation of PGC1α and enhanced nuclear accumulation of NRF2 following oxidative stress. Moreover, we observed pronounced nuclear translocation of PGC1α in MUSE cells, a step essential for the activation of mitochondrial gene programmes. These findings suggest that MUSE cells coordinate mitochondrial removal and replacement more effectively, ensuring continuous energetic competence and redox balance.

The transcriptional choreography underlying this agility appears to be hierarchical. Pharmacological inhibition of PGC-1α with SR-18292 led to a dramatic reduction in mitochondrial biogenesis specifically in MUSE cells, highlighting the important contribution on this pathway. Similarly, NRF2 inhibition with ML385 impaired biogenesis across all cell types, confirming its essential regulatory role. These results argue that PGC-1α appears to play a central and functionally required role in coordinating the oxidative stress response, initiating a broad metabolic re-write that NRF2 subsequently amplifies to fine-tune antioxidant capacity. Such an arrangement reconciles PGC-1α's dual identity as metabolic sensor and chromatin co-activator and explains why attempts to bolster stem-cell resilience by targeting NRF2 alone have yielded inconsistent benefits. Our findings also align with recent reports that PGC1α amplifies MSCs survival in ischaemic models [80,81] and that NRF2 couples redox defence to mitochondrial renewal [82]. By demonstrating their concerted activation in MUSE cells, we extend these observations to a clinically relevant stem-cell subset. Beyond its established role in mitochondrial biogenesis, the PGC1α–NRF2 axis may represent a central regulatory node coordinating multiple adaptive responses observed in MUSE cells. NRF2 is a master regulator of oxidative-stress defence and directly controls the transcription of several antioxidant genes, including SOD1, CAT, and GPX1. In parallel, PGC1α functionally interacts with NRF2 to support mitochondrial adaptation, redox homoeostasis, and cellular stress resistance. Importantly, BER-associated enzymes such as OGG1 can also be regulated directly or indirectly through oxidative-stress-responsive transcriptional networks involving NRF2 and PGC1α. Therefore, the rapid induction of antioxidant enzymes, BER activation, mitophagy, and mitochondrial biogenesis observed in MUSE cells likely reflects an integrated adaptive response coordinated by the PGC1α–NRF2 signalling axis rather than isolated stress-response events.

Importantly, the preservation of global mitochondrial membrane potential in MUSE cells should not be interpreted as evidence that mitochondria targeted for mitophagy remain functionally intact. Rather, our findings support a model in which dysfunctional and depolarized mitochondrial subsets are selectively eliminated through mitophagy, while the remaining healthy mitochondrial network is preserved and replenished through PGC1α-dependent biogenesis. This coordinated ‘remove-and-replace’ mechanism likely explains how MUSE cells maintain overall mitochondrial fitness despite active mitochondrial turnover under oxidative stress conditions.

Conventional MSCs are known to possess moderate antioxidant and regenerative capacities but often undergo mitochondrial dysfunction, senescence, or apoptosis under sustained oxidative conditions. In contrast, pluripotent stem cells typically maintain low ROS levels through glycolytic metabolism and enhanced proteostatic mechanisms, although prolonged pluripotency may also be associated with genomic instability and tumorigenic risk [83]. MUSE cells appear to occupy an intermediate adaptive state in which adult stem-cell properties are combined with robust stress tolerance, efficient mitochondrial quality-control responses, and non-tumorigenic behaviour. Our data suggest that the superior oxidative-stress resilience of MUSE cells does not rely on a single antioxidant mechanism, but rather on the rapid integration of multiple mitochondrial quality-control processes into a coordinated adaptive circuit. In contrast to MSCs and Non-MUSE cells, MUSE cells appear to activate an early hierarchical response in which rapid ROS buffering and DNA repair limit the initial oxidative burden, while PINK1-dependent mitophagy selectively removes dysfunctional mitochondria before irreversible bioenergetic collapse occurs. Simultaneously, PGC1α nuclear translocation and TFAM induction promote rapid mitochondrial replacement, thereby restoring the functional mitochondrial pool. We therefore propose that the distinctive phenotype of MUSE cells emerges from the coordinated synchronization of ROS detoxification, mitochondrial genome protection, selective organelle clearance, and mitochondrial regeneration, rather than from isolated enhancement of individual pathways.

The biological implications of these findings are relevant for understanding stem cell behaviour in oxidative stress–rich environments. Tissues exposed to ischaemia–reperfusion, chronic inflammation or neurodegeneration experience fluctuating redox landscapes that erode cellular bioenergetics and cripple endogenous repair. Our findings provide a mechanistic rationale for the superior outcomes observed when MUSE cells are transplanted into infarcted myocardium, ischaemic brain or ulcerated skin [84]: they arrive with a pre-wired capacity to sense, survive and adapt to oxidative stress. More broadly, the PGC-1α-centred axis offers a tangible target for metabolic engineering. Short-term activation of this pathway could pre-condition other stem-cell populations before infusion, while small-molecule agonists might be deployed *in vivo* to recreate a MUSE-like phenotype in resident progenitors.

These findings expand our understanding of stem cell heterogeneity under oxidative stress and highlight MUSE cells as a biologically relevant model of mitochondrial resilience. The coordinated mitochondrial quality-control strategies identified here may, in the longer term, inform experimental approaches aimed at improving cellular stress adaptation in ROS-rich environments.

This study has several limitations that should be acknowledged. We employed a single, acute H₂O₂ pulse as a controlled oxidative challenge, which allowed high temporal resolution of mitochondrial quality-control dynamics but does not capture the complexity of chronic or spatially heterogeneous ROS exposure *in vivo*. Accordingly, the present findings should be interpreted as a mechanistic analysis of acute oxidative stress adaptation at the cellular level. Although exogenous H₂O₂ represents a broad oxidative insult rather than a mitochondria-specific stressor, several studies have demonstrated that H₂O₂ exposure can secondarily induce mitochondrial ROS accumulation, mitochondrial depolarization, and activation of mitochondrial quality-control pathways through ROS-induced ROS generation mechanisms [71,85–87]. Therefore, mitochondrial adaptive responses likely represent an important downstream component of the cellular oxidative-stress response triggered by H₂O₂ exposure [88]. Although mitochondrial ROS accumulation was evaluated in the present study, future experiments employing mitochondria-specific oxidative-stress inducers such as rotenone or MitoPQ will help further define the specific contribution of mitochondria-derived ROS to MUSE-cell stress resistance [89].

While pharmacological inhibition of PGC-1α and NRF2 provided functional evidence for their involvement in mitochondrial biogenesis and stress resilience, future studies using genetic perturbation and single-cell approaches will be required to fully define the regulatory hierarchy linking antioxidant responses, mitophagy, mitochondrial DNA repair, and biogenesis. In addition, direct evaluation of mitophagy flux would further strengthen the mechanistic interpretation of the observed mitochondrial quality-control response.

Despite these limitations, our data establish that MUSE cells engage a tightly coordinated mitochondrial quality-control programme in response to oxidative stress, characterized by the synchronized removal and replacement of damaged mitochondria. This coordinated response preserves mitochondrial integrity and bioenergetic competence, providing a mechanistic framework for understanding how distinct stem cell subsets adapt to ROS-rich microenvironments.

## Conclusion

In conclusion, our study demonstrates that MUSE cells respond to oxidative stress through a rapid and coordinated mitochondrial quality-control programme integrating antioxidant defence, BER-associated repair, selective mitophagy, and mitochondrial biogenesis. Compared with conventional MSCs and SSEA-3^−^ stromal cells, MUSE cells more efficiently preserve mitochondrial morphology, membrane potential, respiratory function, and mitochondrial genome integrity following oxidative stress exposure.

Our findings support a coordinated ‘remove-and-replace’ model in which damaged mitochondria are rapidly eliminated through mitophagy while the functional mitochondrial network is replenished through PGC1α-driven biogenesis. Rather than relying on a single protective mechanism, MUSE cells appear to synchronize multiple adaptive pathways to preserve mitochondrial fitness and cellular resilience under oxidative stress conditions. Overall, this study identifies mitochondrial quality control as a key determinant of stem-cell stress resistance and provides a mechanistic framework for understanding the remarkable oxidative-stress resilience of MUSE cells.

## Supplementary Material

Supplementary Material Supplementary Files.docx

## Data Availability

The data that support the findings of this study are available from the corresponding author upon reasonable request.
