# Supplementary material for: Coordinated mitochondrial quality control underlies oxidative stress resilience in MUSE stem cells
Source: Redox Rep. 2026 Oct 9;31(1):2698189. doi: 10.1080/13510002.2026.2698189 (PMC13656651; doi:10.1080/13510002.2026.2698189)
Supplement: Supplementary file 1 [file YRER_A_2698189_SM9572.docx]

**Supplementary Figure S1**

**Supplementary Figure S1. Flow cytometry validation of MUSE cell isolation.**

Representative flow cytometry histograms showing SSEA3-positive sorting of MUSE and Non-MUSE cells from the MSC population. Cells were stained with PE-conjugated anti-SSEA3 antibody (red line) or isotype control (black line). MUSE cells (middle panel) displayed a distinct shift in fluorescence intensity compared to the isotype control, confirming positive SSEA3 expression. In contrast, Non-MUSE cells (right panel) showed no difference from the isotype control, confirming SSEA3 negativity. The starting MSC population (left panel) included both SSEA3-positive and -negative subpopulations.

**Supplementary Figure S2**

**Supplementary Figure S2. Validation of subcellular fractionation.**

Western blot analysis confirming the enrichment and purity of mitochondrial (M) and nuclear (N) fractions obtained from MSCs, MUSE, and Non-MUSE cells under basal conditions and after exposure to 300 μM H₂O₂. MT-ND4 was used as a mitochondrial marker and Histone H4 as a nuclear marker, demonstrating selective enrichment and minimal cross-contamination between compartments.

**Supplementary Figure S3**

**Supplementary Figure S3. Nuclear translocation of NRF2 following oxidative stress in MSCs, MUSE, and non-MUSE cells.**

Representative western blot images and densitometric quantification of NRF2 protein levels in cytoplasmic (C) and nuclear (N) fractions under basal conditions and after H₂O₂ exposure. MUSE cells showed a significant increase in nuclear NRF2 accumulation following oxidative stress. In contrast, MSCs and NON-MUSE cells exhibited limited or no significant nuclear NRF2 translocation after H₂O₂ treatment. Data are presented as mean ± SD from 3 independent experiments, *p < 0.05 vs corresponding control condition.

**Supplementary Table 1. List of antibodies**

| **Antibody** | **Code** | **Dilution** |
| --- | --- | --- |
| MFN1 | ELABSCIENCE E-AB- 93135 | 1:1000 |
| MFN2 | ELABSCIENCE E-AB-32025 | 1:1000 |
| DRP1 | ELABSCIENCE E-AB-67035 | 1:1000 |
| FIS1 | ELABSCIENCE E-AB- 93308 | 1:500 |
| Mitobiogenesis cocktail | ABCAM AB123545 | 1:500 |
| PINK1 | ABCAM AB 216144 | 1:500 |
| Ub-S65 | ABCAM AB 303542 | 1:1000 |
| PGC1α | CELL SIGNALING 2178 | 1:400 |
| NRF1 | SANTA CRUZ SC 28379 | 1:500 |
| NRF2 | SANTA CRUZ SC 365949 | 1:500 |
| LAMP1 | CELL SIGNALING 9091 | 1:400 |
| 8-oxo-dg | SANTA CRUZ SC-130914 | 1:1000 |
| ACTB | SANTA CRUZ SC-47778 | 1:8000 |
| H4 | CELL SIGNALING 2592 | 1:5000 |
| GAPDH | SANTA CRUZ SC-365062 | 1:1000 |
| MT-ND4 | SIGMA MABS1994 | 1:1000 |

**Supplementary Table 2. List of primers**

| Primer | Sequence |
| --- | --- |
| *Mito Big Fragment* | Fw: GCCAGCCTGACCCATAGCCATAATAT  Rv: GAGAGATTTTATGGGTGTAATGCGG |
| *Mito Small Fragment* | Fw: CCCAGCTACTACCATCATTCAAGT  Rv: GATGGTTTGGGAGATTGGTTGATG |
| *SOD1* | Fw: TTAATCCTCTATCCAGAA  Rv: TCACAGAATCTTCAATAG |
| *CAT* | Fw: AGAAGTGCGGAGATTCAA  Rv: TTCCTCTGTTCCTCATTCA |
| *GPX1* | Fw: GGAACACCTGATCTTACA  Rv: ACTTTATTAGTGGGGAAAC |
| *OGG1α1* | Fw: AAATCAAGCAGTCAGTTT  Rv:TTTCCATACAAATGTTTCTATT |
| *OGG1α2* | Fw: TATTTCCTGATAACATTATG  Rv: GGTTCTTTAAGGTTCTAA |
| *MUTYHß3* | Fw: TGTTCTGCTGTCTTCATC  Rv: CTTCCTGTGACCACTTCC |
| *MUTYHα3* | Fw: AAGGAGCAGTCCTCTGAA  Rv: GTAGCCTTCAAAGCCTCT |
| *TFAM* | Fw: GACTCAATTCTGTTCTCTTCA  Rv: CTCATTCCTCTATCTCAATCATTA |
| *ACTB* | Fw: CATGTACGTTGCTATCCAGGC  Rv: CTCCTTAATGTCACGCACGA |
| *GAPDH* | Fw: GGAGTCAACGGATTTGGTCGT  Rv: ACGGTGCCATGGAATTTGC |
